# Cross-Linked Microencapsulation of CO_2_ Supercritical Extracted Oleoresins from Sea Buckthorn: Evidence of Targeted Functionality and Stability

**DOI:** 10.3390/molecules25102442

**Published:** 2020-05-23

**Authors:** Corina Neagu, Liliana Mihalcea, Elena Enachi, Vasilica Barbu, Daniela Borda, Gabriela Elena Bahrim, Nicoleta Stănciuc

**Affiliations:** Faculty of Food Science and Engineering, Dunarea de Jos University of Galati, 800008 Galați, Romania; Corina.Neagu@ugal.ro (C.N.); Elena.Ionita@ugal.ro (E.E.); Vasilica.Barbu@ugal.ro (V.B.); gbahrim@ugal.ro (G.E.B.)

**Keywords:** cross-linking, oleoresins, sea buckthorn, microencapsulation, whey proteins, caseins, transglutaminase, functional properties

## Abstract

Oleoresin supercritical extracts from sea buckthorn were microencapsulated in whey proteins isolate and casein, in two states: native (N) and cross-linked mediated by transglutaminase (TG). The encapsulation efficiency showed values higher than 92% for total carotenoids and lycopene. Phytochemicals content was 352.90 ± 1.02 mg/g dry weight (DW) for total carotenoids in TG and 302.98 ± 2.30 mg/g DW in N, with antioxidant activity of 703.13 ± 23.60 mMol Trolox/g DW and 608.74 ± 7.12 mMol Trolox/g DW, respectively. Both powders had an inhibitory effect on α-glucosidase, of about 40% for N and 35% for TG. The presence of spherosomes was highlighted, with sizes ranging between 15.23–73.41 µm and an agglutination tendency in N, and lower sizes, up to 35 µm in TG. The in vitro digestibility revealed a prolonged release in an intestinal environment, up to 65% for TG. Moisture sorption isotherms were studied at 20 °C and the shape of curves corresponds to sigmoidal type II model. The presence of cross-linked mediated aggregates in TG powders improved stability and flowability. Our results can be used as evidence that cross-linked aggregates mediated by transglutaminase applied for microencapsulation of oleoresins have the potential to become new delivery systems, for carotenoids and lycopene, being valuable in terms of their attractive color and biological and bioaccessibility properties.

## 1. Introduction

Sea buckthorn is viewed as a plant with an exceptional functional and biological value, due to the presence of both lipophilic antioxidants (mainly carotenoids and tocopherols) and hydrophilic antioxidants (flavonoids, tannins, phenolic acids, ascorbic acid) in remarkably high quantities [1]. For example, some of the main nutrients, lipids with highly valued fatty acid composition, contribute to the nutritional benefits of sea buckthorn products for consumers. The importance of sea buckthorn is due to well-known positive biological, physiological, and medicinal effects, such as: antioxidative and immunomodulating [2], cardioprotective and antiatherogenic [3,4], antibacterial and antiviral effects [5], healing effect on acute and chronic wounds [6,7], anti-inflammatory [8], antidiabetic [9], anticarcinogenic [10], hepatoprotective and dermatological effects [11] etc.

Several studies were recently focused on the identification of the bioactive compounds profile in sea buckthorn extracts. The main identified components are ascorbic acid, carotenoids, cumaric acid, quinic acid and various phenolics, including proanthocyanidins, gallic acid, ursolic acid, caffeic acid, ferulic acid, catechin and epicatechin derivatives, quercetin, kaempferol, and isorhamnetin glycoside derivatives [3,12,13]. Typically, the extraction and separation of bioactive compounds from the natural sources proceed according to well-established procedures, such as CO_2_ supercritical extraction, which is a green and safe method, enabling one to obtain solvent-free extracts, available for an extensive range of applications [14]. Supercritical fluid extraction, as an alternative to the conventional techniques [15], has emerged in recent decades for the extraction and fractionation of oleoresin from different matrices. For example, supercritical CO_2_ has been used to extract food-grade oleoresins from different plant matrices, including tomato [16,17,18], pumpkin [19,20], watermelon [18], sweet potato tubers [21], rotten onion waste [22], wheat bran [23], spices [14], leaf sea buckthorn [24] and sea buckthorn berries [25], as well as gac [18], or for fractionation of the oleoresins from rosemary ethanolic extract [26].

Sea buckthorn berries are also known as a rich source of carotenoids, such as lutein, zeaxanthin, β-cryptoxanthin, lycopene, γ-carotene, and β-carotene, with the major carotenoids represented by β-carotene followed by lycopene and zeaxanthin [1]. The carotenoids are concentrated in the pulp oil of fresh berries, at variable concentration of 692 to 3420 mg/kg. For example, in sea buckthorn oil, Li and Beveridge [27] identified up to 18 different carotenoids with provitamin A activity (i.e., β-carotene, γ-carotene, β-zeacarotene, β-cryptoxanthin, and sintexanthin) and lutein, accounting for 48 and 14% of the total carotenoids, respectively.

However, when considering carotenoids extracts as attractive ingredients for food or nutraceuticals application, precaution must be taken, because these bioactive compounds are highly susceptible to degradation during processing and storage, due to their sensitivity to temperature, light, and oxygen [28]. Microencapsulation technology provides alternatives for the enhanced stability of sensitive compounds, by creating a protective film layer typically composed by a polymeric material [29]. Therefore, different methods are applied to develop functional barriers between cores and walls, in order to prevent possible physical, chemical or biological interactions. The processes enable the introduction of additional nutrients to food. Fats, oils, flavors, vitamins, enzymes, and dyes are most often microencapsulated [30].

Whey proteins and caseins are milk proteins used frequently as wall materials in microencapsulation, single or in different biopolymeric combinations. Both proteins may be can be enzymatically cross-linked by transglutaminase (TGase), a natural agent of microbial origin recognized as a safe resulting in new structures, with improved encapsulation efficiency of the bioactive compounds [31]. TGase cross-links covalently proteins between the γ-carboxyamide group of glutamine residues and the ε-amino group of lysyl residues [32].

Therefore, the aim of this study was to microencapsulate, by complex coacervation, and freeze-dry oleoresins resulting from the CO_2_ supercritical extraction of sea buckthorn berries, in both native and cross-linked biopolymeric composite materials formed by whey proteins isolate and casein. The two resulting powders, coded N and TG, were characterized for phytochemical profile and encapsulation efficiency, with emphasis on carotenoids, antioxidant activity and in vitro digestibility. The antidiabetic potential was evaluated by the inhibitory effect against α-amylase and α-glucosidase, two of the main enzymes involved in carbohydrate metabolic syndrome. Further, the powders were tested for structure and morphology using the confocal laser scanning microscopy technique. The moisture sorption isotherms provide important information, allowing understanding of the physicochemical changes in powders and prediction of the possible changes that might occur in the stability during storage [33]. The obtained results could bring evidence on how targeted biologically active compounds from plants can be used for increasing their bioaccessibility and stability, with conclusive perspectives in developing food grade ingredients.

## 2. Results

### 2.1. Oleoresin Extraction

It is known that a low moisture content is an important parameter when considering matrices suitable for efficient supercritical CO_2_ extraction of lipophilic compounds. Therefore, the fresh sea buckthorn berries were dried in a convective bed drier at 40 °C, to a dry matter content of 8.53 ± 0.26%. The oleoresins from the extract had a lycopene content of 1921.63 ± 1.57 mg/kg DW, whereas the total carotenoids content was of 4805.76 ± 11.01 mg/kg DW. Lycopene represented approximatively 40% of the total carotenoids content. The total CO_2_ supercritical extraction yield was 121.1 g oleoresin/kg dried berries. Bruno et al. [18] obtained watermelon oleoresins with 7509 mg carotenoids/kg and 3040 mg all-*trans-*lycopene/kg, whereas oleoresins from tomato showed 5217 mg total carotenoids/kg oleoresin, with the highest concentration of 2924 mg all-*trans-*lycopene/kg. Similar values were reported for total carotenoids by Durante et al. [34], who obtained values of 463.1 and 446.2 mg/100 g for pumpkin and tomato oleoresins obtained by supercritical extraction. Devani et al. [22] obtained a high quality of onion oleoresins with a yield of 1012 g/100 g of powder, containing 31 g sulphur/kg oleoresin.

### 2.2. Microencapsulation of Oleoresins

Two microencapsulated powders were obtained in this study, with the main purpose being to valorize the functional and biological potential of carotenoids from oleoresins extracted by supercritical CO_2_ from sea buckthorn. The cross-linked experiments aimed to develop a protein network between whey proteins and casein, able to stabilize the encapsulated compounds. The powders, coded N and TG, were analyzed for encapsulation efficiency, phytochemical and antioxidant activity. In the native state, the encapsulation efficiency showed values of 92.71 ± 0.64% for total carotenoids, and 94.55 ± 0.43% for lycopene. A slightly higher encapsulation efficiency was found in the cross-linked forms of 93.77 ± 0.12% and 96.40 ± 0.72%, respectively. The higher carotenoids loading capacity of whey proteins-casein network produced by enzymatic cross-linking (TG) than non-aggregated network (N) can be associated with their higher surface hydrophobicity, as explained by Li et al. [35] These authors suggested that the surface hydrophobicity of whey proteins isolates was increased by citric acid-mediated cross-linking at a pH value of 7.0, due probably to the higher exposure of the previously naturally buried hydrophobic groups in the proteins’ cores. It seems that the complex network formed by whey proteins and caseins by TGase has more active hydrophobic patches for binding to both carotenes and especially lycopene, thus justifying their slightly higher loading amount and increased efficiency compared to the N.

Both powders showed a satisfactory phytochemicals content of 352.90 ± 1.02 mg/g dry weight (DW) for total carotenoids and 104.61 ± 0.74 mg/g DW for lycopene in case of TG, whereas N presented values of 302.98 ± 2.30 mg/g DW and 69.04 ± 1.45 mg/g DW, respectively. The antioxidant activity values were significantly higher (*p* < 0.05) in TG samples of 703.13 ± 23.60 mMol Trolox/g DW when compared with N (608.74 ± 7.12 mMol Trolox/g DW).

### 2.3. Static In Vitro Digestion

**Figure 1:**
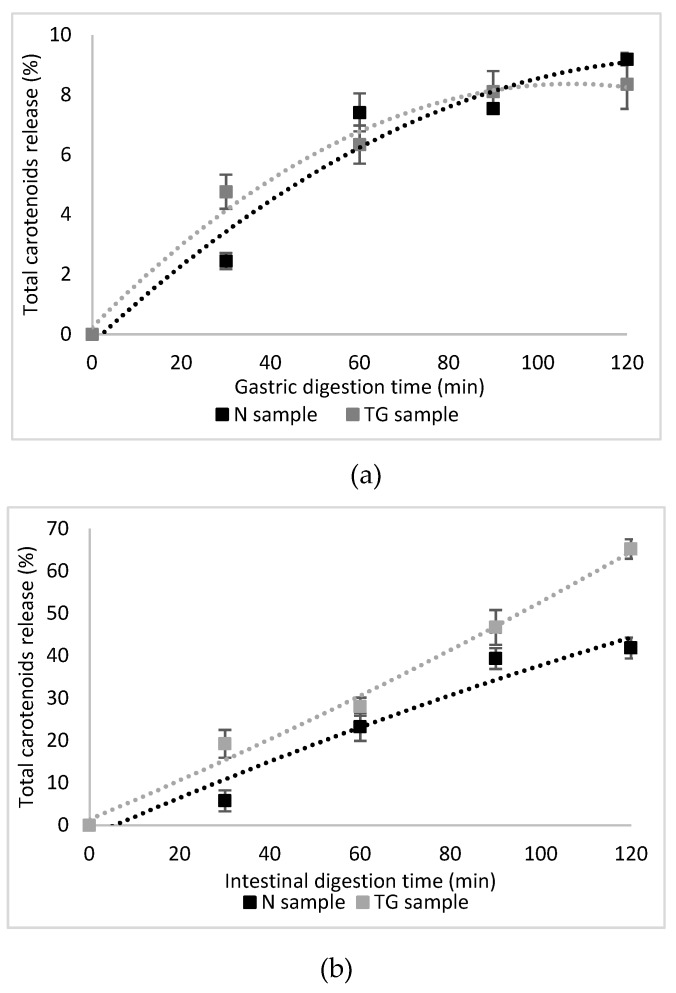
In vitro digestion of total carotenoids in simulated gastric (**a**) and intestinal (**b**) juices.

**Figure 2:**
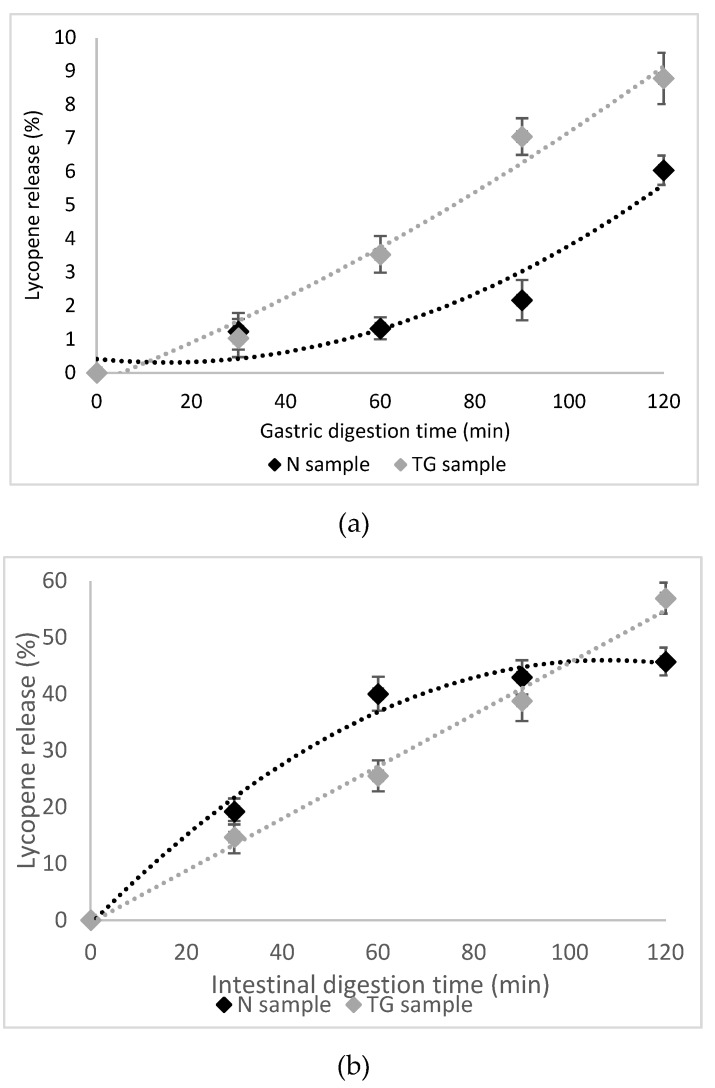
In vitro digestion of lycopene in simulated gastric (**a**) and intestinal (**b**) juices.

A significant proportion of approximatively 70% of β-carotene is lost after the whole digestion as reported by Courraud, Cristol and Avallone [36], with a decrease of 48% during the gastric phase, but with no significant isomerisation in 9- and 13-*cis*-β-carotene. Indeed, carotenoids are more sensitive to acidic than alkaline conditions [37]. In our study, the in vitro release profiles of total carotenoids and lycopene from N and TG during 4 h of digestion are shown in Figure 1 and Figure 2.

After 2 h gastric degradation, about 9% of total carotenoids were released from N in simulated gastric fluid (pH 2.0), whereas the release from TG was about 8% (Figure 1a). A highly protective effect for lycopene was observed in the N sample, where the release was about 6%, compared to the TG sample, where the lycopene release was about 9% (Figure 2a). Therefore, no significant differences (*p* > 0.05) in total carotenoid and lycopene release in the gastric digestion phase was observed for TG sample, while N sample showed a superior protective effect for lycopene.

A sustained-release profile for both total carotenoids and lycopene from N and TG in simulated intestinal fluid was observed and 42% and 65% of total carotenoids was released after 2 h in intestinal juice in N and TG, respectively (Figure 1b). The release of lycopene accounted for approximatively 45% in N and 57% in TG (Figure 2b).

Therefore, it was found that the microencapsulation of carotenoids from sea buckthorn, both in native and cross-linked proteins biopolymeric matrix, was delayed, and a high release into the gastric medium was prevented. The released carotenoids from sea buckthorn extract in the gastric phase was relatively low, up to 10%, but significantly increased in the simulated intestinal juice, up to 65%. TGase mediated crosslinking favored the protection and controlled release of carotenoid compounds in the simulated intestinal juice.

### 2.4. Antidiabetic Activity

Diabetes mellitus is one of the most complex disorders involving chronic hyperglycemia and dyslipidemia, and it is a burden for public health, due to often expensive or unavailable adequate treatment [38]. The pancreatic α-amylase and intestinal α-glucosidase are responsible for hydrolyzing dietary carbohydrates, such as oligosaccharides and disaccharides, into absorbable monosaccharides. In our study, the N and TG showed an inhibitory effect against α-glucosidase of 39.82 ± 0.25% and 34.67 ± 1.00%, whereas a lower effect was found for α-amylase of 14.01 ± 1.42% and 17.95 ± 2.14%, respectively. Wojdyło, Nowicka, and Bąbelewski [38] suggested that the inhibition of these enzymes is specifically useful in the treatment of non-insulin-dependent diabetes, as it slows down glucose release into the bloodstream.

### 2.5. Confocal Laser Scanning Microscopy

The two dyes were applied to the samples, to highlight the binding specificity to the targeted compounds. In our study, DAPI was used to see if the peptides resulting from TGase treatment would bind to the oligonucleotide fragments of the DNA. The fact that the CLSM images did not display any areas colored in blue meant that the aforementioned hypothesis was excluded, so that it was certified that these peptides provided the suitable matrix in which the carotenoids and oleoresin from the sea buckthorn extract were microencapsulated. The scientific literature specifies that congo red is a benzidine-based anionic diazo dye [39], and that it is used as a histological dye which binds to many small proteins or oligo peptides (36–43 amino acids), and for the quantification of β-peptide aggregation [40]. Congo red is also known to display an acidic pH emission peak in the 563–568 nm range (thus emitting in the yellow domain). In our study, in order to promote the coacervation process, the pH of the solutions was adjusted to 3.75, with 1N HCl solution at approximately 40 °C, under constant mechanical stirring at 600 rpm. The two encapsulated powders were structurally analyzed by confocal microscopy. The carotenoids absorption bands are usually located at 448, 476 and 505 nm, whereas their emission bands are around the 630, 685 and 750 nm wavelengths. These values depend on the proportion of the pigments in the plant source [41] recommended the carotenoids’ UV detection at the wavelength of 450 nm, to determine their presence in the analyzed samples.

**Figure 3:**
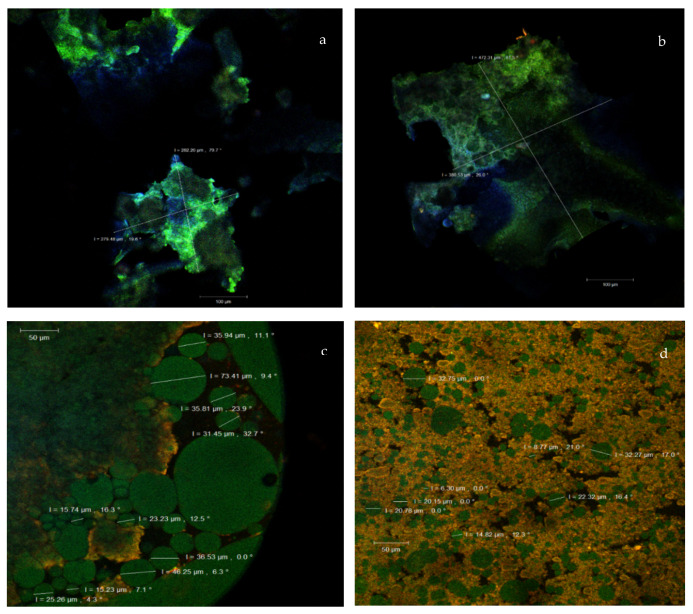
The confocal laser scanning microscopy (CLSM) images of the unstained powders native (N) (**a**) and transglutaminase (TG) (**b**) and the fluorophore dyed powders N (**c**) and TG (**d**).

Our results, supported by Figure 3, showed that the rich in carotenoids and lycopene CO_2_ supercritical extract from sea buckthorn was microencapsulated in the form of spherosomes with the emission being predominantly in the green area, whereas the spherosomes’ biopolymer matrix was shown in yellow. The images of the unstained powders (Figure 3a,b), without any fluorophore addition, highlighted the asymmetric formations of the microencapsulated biocomposites as huge scales. These formations displayed autofluorescence and had sizes between 279.48–282.26 µm for N and were significantly higher (*p* < 0.05) between 380.53–472.31 µm for TG. Furthermore, inside these scales, the spherosomes were formed by the carotenoids trapped in the biopolymer matrix.

The CLSM images of the microscopic samples after the fluorophore staining of the powders with DAPI and congo red are shown in Figure 3c,d. The N sample displayed spherosomes (shown in green), with an agglutination tendency, with different diameters between 15.23–73.41 µm and (Figure 3c). Similar results were obtained by Fu et al. [42], Kirkhus et al. [43], and Li et al. [44], using different microencapsulating biopolymer matrices.

The microencapsulation used for sample TG presented the improved structural appearance of the powder (shown in Figure 3d). After the cross-linking treatment with TGase, the conformation of the wall proteins (WPI and casein) changed, so that the protein exposed new hydrophobic binding sites for carotenoids, ensuring a more efficient encapsulation of the sea buckthorn’s biologically active compounds. Therefore, TG exhibited several spherosomes (shown in green) that were less than 35 µm in diameter. About 80% of these formations were small to medium, more uniform and fixed, stabilized into the biopolymer matrix (shown in yellow) (Figure 3d). Thus, the extensive intra- and interchain cross-linking reactions probably caused the formation of a polymer network which is more stable and useful for various food applications [45].

### 2.6. Moisture Content and Water Activity

Moisture content and water activity are playing a significant role in determining the powders storage stability and foods’ shelf life, as high water content can accelerate lipid oxidation, microbial growth, enzymatic and non-enzymatic reactions, with detrimental effects in foods’ properties. As can be seen in Table 1, the water-activity values of the powders are lower than the threshold value suggested by Labuza and Altunakar [46] (a_w_ = 0.25) for preserving biochemical stability and microbiological safety. The reduced moisture content and water activity guarantee a good stability of both N and TG powders.

### 2.7. Physical Properties of Powders

Bulk density is an important property of powders, since it determines whether the weight of the powder will fit into its package [47]. Bulk and tapped density values of the analyzed powders were low, and between the samples, no significant (*p* > 0.05) differences were observed (Table 1). Similar values were reported by Neves et al. [48] for microparticles containing α-tocopherol. According to Tonon et al. [49], the low bulk density values indicate that more air space is present between particles, and for this reason, the powders are expected to be more sensitive to oxidative degradation during storage.

During reconstitution of powders, porosity (ε) plays an important role. The porosities of powder samples are 0.87 ± 0.18 for powder TG and 0.72 ± 0.39 for powder N. Very close values of porosity were obtained by Premi and Sharma [50] for encapsulated drumstick oil powder, when a high proportion of whey protein concentrate (WPC) was used as carrier (0.58 ± 0.014). The authors mentioned that this may be due to emulsion droplet size that leads to the formation of globule clusters during drying.

The flowability, an important quality parameter of the dried microcapsules, was determined based on Hausner ratio and Carr index. The powders with Carr index values between 11–15% and Hausner ration values between 1.12–1.18 are considered as powders with good flowability [51]. The N and TG powders have higher values for Carr index and Hausner ratio (Table 1) than the ranges indicated by Lebrun et al. [51], so, according to this classification, the flowability can be considered as poor to very poor. The possible reason for the poor flowability of these powders may be to the presence of large size microcapsules and low moisture content, which decrease the cohesion and frictional force but also could be due to the high content of surface oil which causes particles to stick together and increase the resistance to flow [50]. It should be noticed, though, that significantly higher values (*p* < 0.05) were obtained for TG powder compared to N powder, which suggests that the addition of TGase could improve powder flowability.

#### 2.7.1. Hygroscopicity

Hygroscopicity (water adsorption) is a critical quality parameter for encapsulated oil, since the presence of water can affect the powder flowability and cause lipid oxidation [50] and is mainly influenced by physical parameters such as surface area and particle morphology. According to Nurhadi at al. [52], powders with <20% hygroscopicity are regarded as not very hygroscopic. The values obtained in this study were less than 6% (Table 1), suggesting the good stability of both powders, while significantly lower values (*p* < 0.05) were obtained for TG 4.76 ± 0.09% powder. Thus, it can be considered that the TGase presence could improve microcapsules stability by lowering hygroscopicity. Low values of hygroscopicity can be attributed to the method applied for micoencapsulation and the carrier agents used. According to Man et al. [53], during freeze drying, large particles are formed. A large particle size means low exposed surface area and low water absorption [47,54]. The presence of WPC in the encapsulation matrix can reduce the hygroscopicity of the powder, due to its film formation characteristics [55]. The same observation was reported by Tonon and al. [49], Adhikari et al. [56] and Moghbeli et al. [57] A powder with low hygroscopicity is desirable, providing a better protection in the food matrix when exposed to the environment, due to a lower moisture absorption compared to the one with high hygroscopicity [58].

#### 2.7.2. Sorption Isotherms

The sorption isotherms can provide data about the shelf life stability of a given food commodity. Dried microcapsules are physically stable at moisture contents below 20%. However, high relative humidity conditions can cause a reduction in the glass transition temperature, due to plasticization by water absorption [59]. In porous food products, water transfer is complex, with different mechanisms occurring, vapor diffusion in air-filled pores as a result of vapor pressure gradients and movement of liquid due to capillary action [60].

During storage, modification of the moisture content and water activity of powders as a result of changes in temperature and environmental moisture content might also change the properties of powders, flowability being primarily affected [60] and the controlled release of the inner structure that could also be compromised by exposing the sensitive oleoresins to oxidation. Sorption isotherms provide information on the powders’ stability that allows one to take protection measures against detrimental changes during storage. In the current study, both powders (TG and N), after 3 weeks of storage at relative humidity of 97%, acquired a similar moisture content of 20.57% for TG powder and of 20.94% for N, very close to the threshold value that could affect microcapsules stability. Thus, in order to protect the microcapsules, the powders is recommended to be stored at a lower relative humidity than 94%.

**Figure 4:**
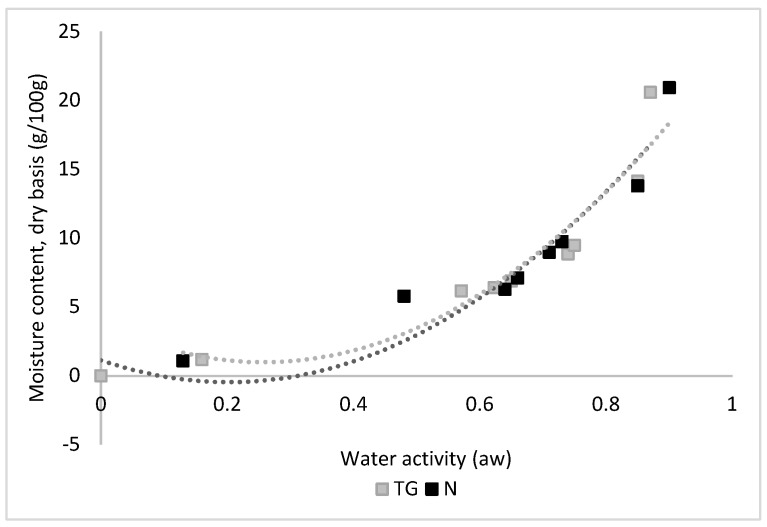
Water sorption isotherms (20 °C) of the powders (N and TG).

The water absorption isotherms for powders with oleoresin at 20 °C are presented in Figure 4. From the graph, it can be observed that the shape of the curves corresponds to sigmoidal type II, according to the Brunauer classification [61]. A similar type of curves was observed by many researchers for powders with whey proteins [48,62,63]. Figure 4 shows the water content increased with the increase in a_w_ for both powders. The reason for this behavior could be the hydrophilic nature of the protein present in the powders analyzed [64,65].

GAB, Halsey, Oswin, Chung and polynomial models were fitted to the experimental sorption data. The fitting parameters were determined by a non-linear regression analysis and are presented in Table 2.

The goodness of fit was evaluated using statistical parameters *R^2^* and *RMSE*, which indicate the fitting ability of models to a data set. Models with *R^2^* values higher than 0.980 or *RMSE* values lower than 15% can be considered acceptable [66]. The equations which have a high *R^2^* and low *RMSE* values are Halsey followed by the GAB model. GAB provides the advantage of estimating the monolayer humidity, a parameter that could be further used in estimating other physical properties.

GAB monolayer (*X_m_*) values were 0.024 and 0.029 kg H_2_O/kg dry matter for TG and N powders, respectively (Table 2). Similar values for *X_m_* were reported for spray dried dairy powders containing intact whey proteins by Kelly et al. [63]

*X_m_* the monolayer moisture content, (kg H_2_O/kg DW), *C* the GAB model parameter, *K* the GAB model parameter, *a* is the constant, *k* the constant, *a_w_* the water activity, *b* the constant.

According to Andrade et al. [67], the monolayer value can indicate the optimum quantity of moisture in dried foods associated with negligible loss in product quality in terms of aroma retention, color and biological value. The K-value involves interactions between water molecules and the adsorbent (powders) in the multilayer. According to Lewicki [68], the GAB model describes sigmoidal-type isotherms well, when K values fall between 0.24 and 1. For the N and TG powders obtained, the K values fell within this range, being 1 for TG powder and 0.95 for N powder, respectively.

The *S*_0_ values of freeze dried powders are 84.39 m^2^/g for powder N and 102.82 m^2^/g for TG powder, respectively. Labuza [69] indicated that *S*_0_ values of food products are within the range of 100–250 m^2^/g. At the equilibrium moisture content, the pores radius was smaller for the N powder than for the TG powder (Table 2), however at higher humidity than 0.14 kg water/kg DW, the TG powder had a smaller pore radius (1.17 nm—TG and 1.29 nm—N). Considering the entire moisture content range studied (0.012–0.21 kg water/kg DW), it can be noticed that both powders remained smaller than 2 nm and they can be considered according to the International Union of Pure and Applied Chemistry (IUPAC) micropores.

## 3. Materials and Methods

### 3.1. Materials

#### Sea Buckthorn Berries

Mara fresh and ripe sea buckthorn berries (*Hippophae rhamnoides* L.) were purchased from a local producer (Biofarmnet from Ialomita city, Romania). The sea buckthorn berries (SBT) were manually separated from leaves and dried in a convective bed drier at 40 °C to a constant weight. Dried berries were grinded in a pilot mill (model MC 12, Stephan, Germany) and used for supercritical CO_2_ oleoresin extraction.

### 3.2. Chemicals

The HPLC analytical-grade hexane, acetone, methanol and analytical grade [2,20 azinobis (3-ethylbenzothiazoline-6-sulfonic acid) diammonium salt] (ABTS) and 6-Hydroxy-2,5,7,8-tetramethylchromane-2-carboxylic acid (Trolox), magnesium carbonate were obtained from Sigma Aldrich (Steinheim, Germany). Whey proteins isolate and casein with 94% protein content were purchased from Fonterra (Clandeboye, New Zealand).

### 3.3. Methods

#### 3.3.1. Extraction of Oleoresins from Sea Buckthorn Berries

The supercritical CO_2_ extraction method was employed to obtain oleoresin from the dried and grinded sea buckthorn berries, as described by Mihalcea et al. [25]. The extraction parameters were 27.6 MPa, 34.51 °C and 82.0 min for pressure, temperature and time of extraction, respectively. The extraction yield was expressed as the ratio between the weight of extracted oleoresin and the weight of matrices loaded into the extractor vessel. The obtained extract was packed in brown plastic jars to protect from light, and stored at −18 °C for further analysis.

#### 3.3.2. Phytochemical Profile of the Extract

The obtained extract was characterized in terms of lycopene and total carotenoid contents by the spectrophotometric method. An amount of 0.1 g of extract was dissolved in 10 mL of a mixture of *n*-hexan:acetone (ratio of 1:1), in a volumetric flask. The absorbance was measured at 470 nm and 503 nm. The amount of lycopene/total carotenoids was calculated according to the following equation:

(1) $$Lycopene\;\left(mg/g\right)=A\cdot M_{W}\cdot D_{f}/\left(M_{a}\cdot L\right)$$

(2) $$Total\;carotenoids\;\left(mg/g\right)=A\cdot M_{W}\cdot D_{f}/\left(M_{a}\cdot L\right)$$

with: *A*—absorbance at 503 nm and 470 nm, respectively, *M_w_*—molecular weight for lycopene and β-carotene (536.873 g·mol^−1^ and 536.8726 g·mol^−1^, respectively), *D_f_*—sample dilution rate, *M_a_*—molar absorptivity for lycopene and β-carotene in *n*-hexan (3450 L·mol^−1^·cm^−1^ and 2500 L·mol^−1^·cm^−1^, respectively), and *L*—cell diameter of the spectrophotometer (1 cm).

#### 3.3.3. Microencapsulation of Oleoresins

The microencapsulation of oleoresins was performed by complex coacervation, as described by Mihalcea et al. [25] Whey proteins isolate (WPI) and casein were dissolved in distilled water at a ratio of 1:1 (*w*/*w*). The colloidal solution was allowed to mix on a magnetic stirrer, until complete hydration at 650 rpm. Prior to addition of oleoresin, half of the mixture was divided and enzymatically cross-linked with Tgase, in a ratio of 1:10 (*w*/*w*), at 40 °C for 2 h. After cross-linking, 50 g of oleoresin was added to 250 mL of biopolymeric colloidal solutions, by stirring to form a coarse emulsion. The coarse emulsion was then homogenized using an Ultra Turrax mixer (IKA T18 basic), at 10,000 rpm for 5 min. In order to promote coacervation, the pH of the solutions was adjusted to 3.75, with 1N HCl solution at approximately 40 °C, under constant mechanical stirring at 600 rpm. The reaction mixture was then allowed to cool in ice bath under stirring, and stored at 4–6 °C overnight to promote decantation. After separation, the coacervates were freeze-dried (CHRIST Alpha 1–4 LD plus, Osterode am Harz Germany) at −42 °C, under a pressure of 10 Pa for 48 h. Afterwards, the powder was collected and packed in metallized bags, and kept in the refrigerator at 4 °C until further analysis.

#### 3.3.4. Encapsulation Efficiency

The encapsulation efficiency was employed as described by Souza et al. [70], considering the amount of total and surface bioactives in powder. For total bioactives content, in terms of lycopene and total carotenoinds, about 250 mg of the powder was dissolved in 6 mL of 10% NaCl:methanol (ratio of 1:1), followed by sonication for 30 min to break the microcapsules. A volume of 30 mL hexan:acetone mixture (1:1) was further added. The samples were centrifuged at 6000× *g* for 10 min. Absorption was read in a spectrophotometer in the visible wavelength 470 nm and 503 nm. The same procedure was used for the surface, skipping the addition of 6 mL of 10% NaCl:methanol (ratio of 1:1) and sonication. Encapsulation efficiency was calculated using Equation (3):

(3) $$Encapsulation\;efficiency\;\left(\%\right)=\frac{Total-Surface}{Total}\times 100$$

#### 3.3.5. Antioxidant Activity

The ABTS^+^ radical’s method was employed as described by Hashemi et al. [71] A volume of 2.85 mL of the ABTS solution (absorbance of 1.12 ± 0.02 at 734 nm) and 0.15 mL of the extract or powder solution were allowed to react for 2 h in a dark room. The absorbance was determined at 734 nm. The ABTS^+^ antioxidant activity of the samples was expressed as mM Trolox/g DW of sample, based on the calibration curve.

#### 3.3.6. Antidiabetic Activity

The method described by Wang et al. [72] for the inhibitory effect against α-amylase and α-glucosidase. Briefly, for α-amylase inhibitory effect equal volumes (200 μL) of powders (10 mg dry weight equivalent/mL in 0.1 M phosphate buffer, pH 6.9) and 1% (*w/v*) starch solution were incubated in Eppendorf tubes at 25 °C for 10 min. A volume of 200 μL of α-amylase (1 mg/mL in 0.1 M phosphate buffer, pH 6.9) was added to each tube, and the reaction mixtures were further incubated at 25 °C for 10 min. The reaction was stopped with the addition of 1 mL of 3,5-dinitrosalicyclic acid reagent solution and incubated at 100 °C for 5 min. Then, the mixtures were diluted with 5 mL of MilliQ water and absorbance was measured at 540 nm.

For α-glucosidase, the procedure involved the addition of equal volumes (50 μL) of powders (10 mg dry weight equivalent/mL in 0.1M phosphate buffer, pH 6.9) and enzymes solution (1 mg/mL in 0.1M phosphate buffer, pH 6.9), followed by incubation at 37 °C for 20 min. Furthermore, 20 μL of 25 mM *p*-nitrophenyl-α-d-glucopyranoside in phosphate buffer 0.1M, pH 6.9 was added and incubated at 37 °C for 40 min in darkness. Acarbose was used as positive control. The amount of *p*-nitrophenol released was quantified at 405 nm.

Inhibitory effect was evaluated as (Equation (4)):

(4) $$Inhibitory\;effect\;\left(\%\right)=\frac{A_{C}-A_{S}}{A_{c}}\times 100$$

where *A_C_* and *A_S_* were the absorbance of the control and sample, respectively.

#### 3.3.7. Static In Vitro Digestion

The method described by Oancea et al. [73] was used to assess the in vitro digestibility of carotenoids from powders. The digestion model was composed of gastric and intestinal simulated juices. The samples (10 mg/mL in phosphate buffer) were digested sequentially at 37 °C in an SI e 300R orbital shaking incubator (Medline Scientific, Chalgrove, UK), at 100 rpm, as follows: addition of 10 mL of powder to 10 mL of gastric juice (4 mg/mL pepsin in 0.1 M HCl buffer, pH 2.0) and mixing for 2 h and intestinal digestion, by mixing 5 mL from gastric digestion with 5 mL of intestinal simulated juice (5 mg/mL pancreatin in 0.1 M sodium bicarbonate, pH 8.0) and mixing for 2 h. Aliquots (1 mL) of each fraction were collected, centrifuged, and diluted with 2 mL of *n*-hexane and the absorbance was read at 503 nm and 470 nm, respectively. The samples were prepared in triplicate (n = 3).

#### 3.3.8. Confocal Laser Scanning Microscopy

In order to observe the structure and the morphology of the sea buckthorn encapsulated powders, a confocal analysis was performed on a Zeiss Axio Observer Z1 (Carl Zeiss Microscopy GmbH, Köln, Germany) confocal inverted microscope. The LSM 710 microscope consists of several laser scanning systems: diode laser (405 nm), Ar laser (458, 488, and 514 nm), DPSS (561 nm pumped solid-state diodes), and HeNe laser (633 nm). The distribution of the biologically active compounds from sea buckthorn into the complex matrix was observed using a 20× apochromatic objective and the 0.6 magnification. Both of the obtained powders were analyzed in their native state and also fluorescently labeled with 4′,6-diamidino-2-phenylindole (DAPI) (1 µg/mL) and Red Congo (40 µM), in a ratio of 3:1:1. The captured confocal images of the encapsulated powders were analyzed with the ZEN 2012 SP1 software (Black Edition, Carl Zeiss Microscopy GmbH, Jena, Germany).

#### 3.3.9. Moisture Content and Water Activity (a_w_)

The moisture content of the freeze dried powder samples was determined using the reference gravimetric method [74], while the water activity was established using a water activity meter (fast lab water activity meter; GBX, Loire, France), based on the chilled mirror dew point technique.

#### 3.3.10. Hygroscopicity

Hygroscopicity determination was based on the method applied by Caparino et al. [75], with some modifications. Briefly, two grams of each powder were placed in a Petri dish inside a sealed humidity jar, with NaCl saturated solution (75.5% humidity), and stored at 25 °C for 7 days. Hygroscopicity (HG, %), was reported as g of adsorbed moisture per 100 g dry solids (g/100 g).

### 3.4. Selected Physical Properties of Powders

#### 3.4.1. Bulk and Tapped Density

The bulk density of the powders was measured following the procedure described by Crouter and Briens [76], with some modification. One gram of each powder (N and TG) was weighed and placed in a 25 mL graduated cylinder. The volume was read directly from the cylinder and the bulk density value was determined as the ratio of mass of the powder and the volume occupied in the cylinder. To determine the tapped density, the cylinder was tapped manually from a height of 10 cm, until negligible difference in volume between succeeding measurements was observed (V_0_). Tapped density (g/mL) was calculated with Equation (5):

(5) $$\rho_{T}=\frac{m_{0}}{V_{0}}$$

#### 3.4.2. Particle (True) Density

To determine the particle density, a method described by Premi and Sharma [50] was applied. The particle density (g/mL) was calculated with Equation (6):

(6) $$\rho_{p}=m_{0}/V_{1}$$

where, *m*_0_—is the mass of powder (g), *V*_1_—is the toluene volume level (mL)

#### 3.4.3. Porosity

The relationship between bulk density (*ρ_B_*) and particle density (*ρ_p_*) described by Equation (7) was used to determine the porosity of powder samples [77]:

(7) $$Ɛ=1-\left(\frac{\rho_{B}}{\rho_{p}}\right)$$

To characterize the powder flow properties, the Hausner Ratio (HR) and Carr Index (CI) were calculated (Equations (8) and (9)) [76]:

(8) $$CI\;=\frac{\mathrm{tapped}\;\mathrm{density}\;-\;\mathrm{bulk}\;\mathrm{density}\;}{\mathrm{tapped}\;\mathrm{density}}\times 100\left(\%\right)$$

(9) $$\mathrm{HR}=\frac{\mathrm{tapped}\;\mathrm{density}}{\;\mathrm{bulk}\;\mathrm{density}}$$

#### 3.4.4. Sorption Isotherms

The adsorption isotherms of the freeze-dry powders were determined at 20 °C by the static method, using seven saturated salt solutions (MgCl_2_, KI, NaCl, NH_4_Cl, (NH_4_)_2_SO_4_, KNO_3_, K_2_SO_4_), at a_w_ values ranging from 0.33 to 0.977 [78]. In each jar with saturated solution, a glass capsule with about 0.5 g of sample was placed. At water activity higher than 0.65, phenol was placed inside the desiccators, to prevent the microbial spoilage of the material. The samples were weighed initially, after 3 days, and then daily, until the difference between two consecutive weights was less than 0.001 g. The water adsorption kinetics curves were obtained at equilibrium moisture contents and reported as g water/g dry weight. To correlate the equilibrium moisture contents with the relative humidity of the environment GAB, Halsey, Polinomial, Chung and Oswin models were used (Table 1). The models were fit to the experimental data by nonlinear regression (SAS software, version 9.1, Cary, NC, USA). As a basis for model accuracy in predicting the response, root mean square error (RMSE) value was calculated for all the tested models (Equation (10)) [79]:

(10) $$RMSE=\sqrt{\frac{\sum_{i=1}^{i}{\left(y_{exp}\left(t_{i}\right)-y\left(t_{i},p_{is}\right)\right)}^{2}}{n_{t}-n_{p}}}$$

where, *y_exp_*(*ti*) denotes the experimental observations, *y*(*t_i_,p_is_*) the predicted values, *n_t_* the total number of data points and *n_p_* the number of estimated model parameters. Standard deviation of the estimated parameters was also a good indicator for the contribution of the individual parameters to the model.

#### 3.4.5. Specific Surface Area of Sorption

The surface area of sorption (*S*_0_) plays an important role in determining the bond water of a particulate material, and is determined from the monolayer moisture *X_m_* obtained from the GAB model (Equation (11)) [80]:

(11) $$S_{0}=M_{0}\frac{1}{M_{w}}N_{0}A_{H_{2}O}$$

where, *M*_0_—is the monolayer moisture content (GAB model), *M_w_—*molecular weight of water (kg/mol), *N*_0_ is the Avogadro number (6 × 10^23^ molecules/mol), *A_H2O_* is the area of a water molecule (1.06 × 10^−19^ m^2^/molecule).

#### 3.4.6. Effective Pore Size of Sorption

The Kelvin equation (Equation (12)), which was primarily applied in the condensation region of the isotherm, was employed to calculate the critical pore size [81]:

(12) $$r_{c}=-\frac{2\sigma V_{m}}{RTln\left(a_{w}\right)}$$

where, *r_c_* is the critical pore size (m), *σ* is surface tension of water (N/m), *V_m_* is molar volume of adsorbate in bulk liquid state (m^3^/mol), *R* is universal gas constant (8.314 × 10^−3^ kJ/mol·K), *T* is absolute temperature (K).

The Halsey equation (Equation (13)) was applied to calculate the adsorbed water multilayer thickness [81]:

(13) $$t=0.345{\left[\frac{-5}{ln\left(a_{w}\right)}\right]}^{1/3}$$

where, *t* is the adsorbed water multilayer thickness (nm).

The effective pore size of sorption (*r_p_*) may be obtained by the sum of critical radius and the multilayer thickness (Equation (14)) [81]:

(14) $$r_{p}=r_{c}+t$$

### 3.5. Statistical Analysis

All experiments and measurements were carried out in triplicate. The results are expressed as mean ± standard deviation. The results were compared statistically using ANOVA and Tukey tests (Minitab 18 Statistical Software). Differences between groups were considered significant at *p* < 0.05.

## 4. Conclusions

In summary, the biopolymeric matrix represented by milk proteins was used to microencapsulate oleoresins obtained by supercritical fluid extraction from sea buckthorn, in different structural and conformational states, namely as native and as enzymatically cross-linked via transglutaminase mediated reaction. Both states were successfully employed to load supercritical extracted oleoresins from sea buckthorn by complex coacervation and freeze-drying, in terms of total carotenoids and lycopene, yielding fine and intense yellow powders. Both powders showed a remarkable content in total carotenoids and lycopene, antioxidant activity and antidiabetic potential. However, the cross-linked aggregates favored the release of the carotenoids in the intestinal simulated environment, probably due to the specific structural and morphological characteristics. In this regard, the cross-kinked mediated microencapsulation led to a more fine powder, with microparticles having significantly smaller diameter, which improved the release profile. Moisture sorption isotherms were studied at 20 °C and the best models fit to the experimental data were Halsey and GAB. The shape of curves corresponds to sigmoidal type II similar with that of powders with whey protein carrier agent. Presence of cross-linked mediated aggregates improves microcapsules stability and flowability.

In general terms, this research suggested that the enzymatically mediated cross-linked aggregates could be promisingly employed in food applications, as targeted delivery vehicles for carotenoids as bioactive ingredients.

## Figures and Tables

**Table 1:** Physical properties of powders.

Physical Properties	Variants	
	N	TG
Moisture content (g water/100 dry solids)	1.1 ± 0.17 ^a^	1.2 ± 0.07 ^a^
Water activity	0.13 ± 0.03 ^a^	0.12 ± 0.03 ^a^
Bulk density (g/cm^3^)	0.14 ± 0.00 ^a^	0.13 ± 0.01 ^a^
Tapped density (g/cm^3^)	0.19 ± 0.01 ^a^	0.21 ± 0.01 ^a^
Particle density (g/cm^3^)	0.45 ± 0.39 ^a^	0.46 ± 0.05 ^a^
Porosity	0.72 ± 0.39 ^a^	0.87 ± 0.18 ^a^
CI, %	27.70 ± 1.84 ^b^	37.76 ± 0.07 ^a^
HR	1.38 ± 0.04 ^b^	1.61 ± 0.00 ^a^
Hygroscopicity,%	5.79 ± 0.37 ^a^	4.76 ± 0.09 ^b^

Columns that do not share a letter are significantly different (*p* < 0.05).

**Table 2:** Estimated values of the parameters and statistical coefficients for the mathematical models of GAB, Halsey, Chung, Polinomial, and Oswin.

Parameter	TG	N
GAB $X_{\mathrm{eq}}=\frac{X_{m}{\mathrm{CKa}}_{w}}{\left(1-{\;\mathrm{Ka}}_{w}\right)\left(1-{\;\mathrm{Ka}}_{w\;}+{\;\mathrm{CKa}}_{w}\right)}$		
X_m,_ (kg H_2_O/kg dry matter) C K	0.024 8.96 1.01	0.029 7.47 0.96
R^2^	0.98	0.99
RMSE	1.88	1.36
Halsey $\;a_{w}=e^{\frac{a}{M^{n}}}$		
A b	2.38 0.90	2.8 0.95
R^2^	0.97	0.98
RMSE	0.086	0.059
Oswin $X_{\mathrm{eq}}=\;a{\left[\frac{a_{w}}{\left(1-{\;a}_{w}\right)}\right]}^{b}$		
A b	4.09 0.80	4.82 0.65
R^2^	0.99	0.99
RMSE	1.31	0.86
Chung $M\;=\;a\;+\;\mathrm{bln}\left(-{\mathrm{lna}}_{w}\right)$		
a	2.73	3.32
b	−6.65	−6.24
R^2^	0.84	0.89
RMSE	2.55	2.14
Polinomial $M\;=\;a\;+{\;\mathrm{ba}}_{w}+{\;\mathrm{ca}}_{w}^{2}$		
a	4.94	3.79
b	−29.80	−21.72
c	50.55	42.13
R^2^	0.90	0.92
RMSE	2.18	2.02
